# Trophic convergence drives morphological convergence in marine tetrapods

**DOI:** 10.1098/rsbl.2014.0709

**Published:** 2015-01

**Authors:** Neil P. Kelley, Ryosuke Motani

**Affiliations:** 1 Department of Paleobiology, National Museum of Natural History, Smithsonian Institution, PO Box 37012, Washington, DC 20013, USA; 2 Department of Earth and Planetary Sciences, University of California, Davis, 1 Shields Avenue, Davis, CA 95616, USA

**Keywords:** marine mammal, marine reptile, convergent evolution, feeding adaptation, functional morphology

## Abstract

Marine tetrapod clades (e.g. seals, whales) independently adapted to marine life through the Mesozoic and Caenozoic, and provide iconic examples of convergent evolution. Apparent morphological convergence is often explained as the result of adaptation to similar ecological niches. However, quantitative tests of this hypothesis are uncommon. We use dietary data to classify the feeding ecology of extant marine tetrapods and identify patterns in skull and tooth morphology that discriminate trophic groups across clades. Mapping these patterns onto phylogeny reveals coordinated evolutionary shifts in diet and morphology in different marine tetrapod lineages. Similarities in morphology between species with similar diets—even across large phylogenetic distances—are consistent with previous hypotheses that shared functional constraints drive convergent evolution in marine tetrapods.

## 1. Introduction

Terrestrial vertebrates have repeatedly readapted to marine life since their ancestors originally left the water over 300 Myr ago [1–4]. These habitat shifts, and their attendant changes in diet and morphology, have resulted in increasing ecological and anatomical disparity within many secondarily marine tetrapod lineages. In contrast to this pattern of increasing disparity within lineages, distantly related marine tetrapod species have independently adapted towards similar lifestyles and morphologies [5,6], providing textbook illustrations of evolutionary convergence.

Contrasts in the types and distribution of trophic resources between terrestrial and marine environments and fundamental differences in the physical properties of water and air constrain foraging modes among marine tetrapods [6]. Thus, apparent similarities in marine tetrapod skull and tooth morphology have often been interpreted to reflect convergent adaptation towards specific diets and feeding modes. Qualitative comparison of marine tetrapod morphology is strikingly suggestive of convergence in many cases, but this approach can be misleading [7]. Here, we provide a quantitative approach for investigating ecomorphological convergence across living marine tetrapod clades.

## 2. Material and methods

Diets of 69 marine mammal and reptile species were tabulated using approximate proportion of diet (0–1) within eight dietary categories used by Pauly *et al*. [8], plus an additional category ‘plants’ (see the electronic supplementary material for detailed methods and table S1 for dietary proportions and sources). The enormous size and highly derived morphology and feeding modes of baleen whales [9] led us to exclude mysticetes in the present analysis. We used Ward's minimum variance clustering to group species with similar diets and identify feeding guilds. The strength of clusters was evaluated with confidence intervals calculated from 10 000 multiscale bootstrap resampling. Cluster analysis was carried out using R v. 3.0.3 [10], and bootstrapping *p*-values were calculated using the package pvclust [11].

Seventeen functionally important skull and jaw measurements were collected from each species, and 12 tooth measurements were collected for species with complete dentition (see the electronic supplementary material, figure S1 for a complete list of measurements). Measurements were taken with digital callipers (0.01 mm accuracy). Measurements longer than 300 mm were taken with analogue callipers or a tape measure. See the electronic supplementary material (table S2) for a complete list of specimens used in this study.

We used linear discriminant analysis (LDA) to test the ability of these measurements to discriminate between the dietary categories identified in the cluster analysis. All measurements were log-transformed prior to LDA. We calculated LDA scores using three combinations of variables: (i) skull and jaw measurements only; (ii) tooth measurements only; and (iii) cranial and tooth measurements combined. The latter two analyses included fewer species (54 of 69) owing to the exclusion of species with reduced or absent dentition. LDA was conducted using the mass package [12] and R v. 3.0.3 [10]. We avoided ‘correcting’ statistical analyses for phylogeny (e.g. independent contrasts), because we are specifically interested in the degree to which similarities in diet and morphology transcend deep phylogenetic divergences between lineages that adapted to marine life independently.

We assembled a time-calibrated phylogeny of the 69 species from previously published phylogenetic analyses (see the electronic supplementary material, figure S2) and timetree.org to trace the history of trophic diversification within clades. Trophic groups identified by cluster analysis were mapped onto this tree and parsimony was used to reconstruct hypothetical dietary habit of internal nodes using Mesquite v. 2.75 [13].

## 3. Results

**Figure 1.**
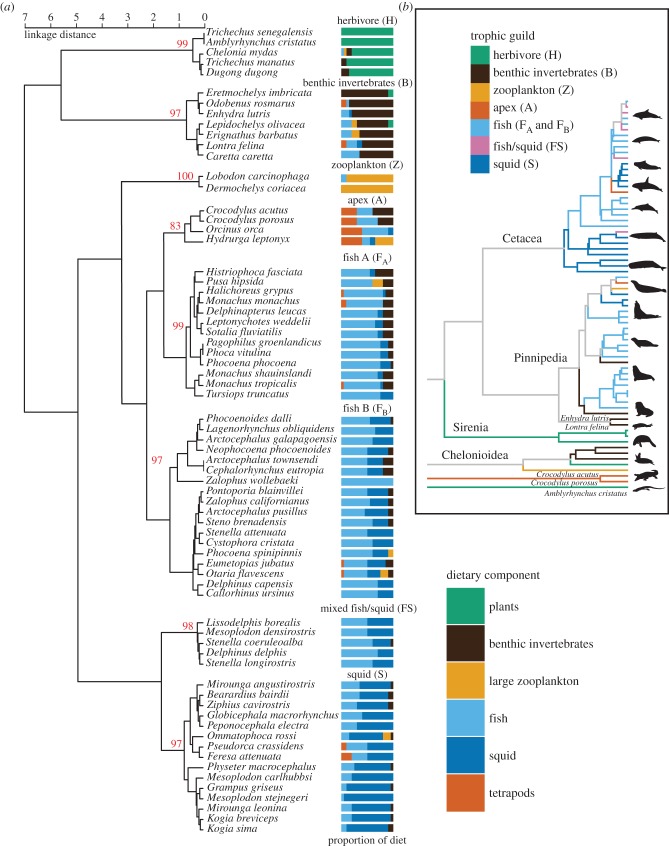
(*a*) Cluster analysis of dietary data for marine tetrapods. Red numbers show bootstrap confidence intervals. (*b*) Summarized phylogenetic distribution of trophic guilds across all species in this analysis (see the electronic supplementary material for detailed phylogeny and sources). Silhouettes in (*b*) by Chris Huh, Vince Smith, Steven Traver from phylopic.org and the authors.

Cluster analysis of dietary data resolved well-supported groupings of species sharing similar diets (figure 1*a*), representing as many as eight distinct trophic guilds. In roughly increasing trophic level, these are: (i) herbivores (H); (ii) benthic invertebrate specialists (B); (iii) zooplanktivores (Z); (iv and v) two distinct groups that feed primarily upon fish (F_A_ and F_B_), but differ in the relative proportion of types of fish consumed (demersal, versus schooling pelagic); (vi) a group that feeds on a roughly equal proportion of mesopelagic fish and cephalopods (FS); (vii) squid specialists (S) and (viii) apex predators (A), which consume a significant fraction of tetrapod prey in addition to fish and invertebrates. These guilds were well supported by thousand-replicate bootstrap confidence intervals above 95%, except for the apex guild (CI = 83%).

Each trophic guild identified by cluster analysis of dietary data includes phylogenetically disparate species (figure 1*b*), although many subclades (e.g. families and subfamilies) are characterized by a similar diet. Of the four largest marine clades considered here—odontocetes, pinnipeds, sirenians and chelonioids—all but sirenians span multiple trophic categories. Five individual lineages that have invaded marginal marine environments more recently (*Crocodylus acutus*, *C. porosus, Enhydra, Lontra felina*, *Amblyrhynchus*) also span a range of trophic guilds.

LDA of skull and tooth data provided moderate-to-high discrimination (80–100%) among the eight trophic guilds, with the exception of the two trophic clusters with fish-dominated diets (F_A_ and F_B_), which overlapped substantially in morphology. Merging these two trophic guilds into a single fish-dominated dietary category substantially improved LDA outcomes, and this approach was used for the LDA results presented below.

**Figure 2.**
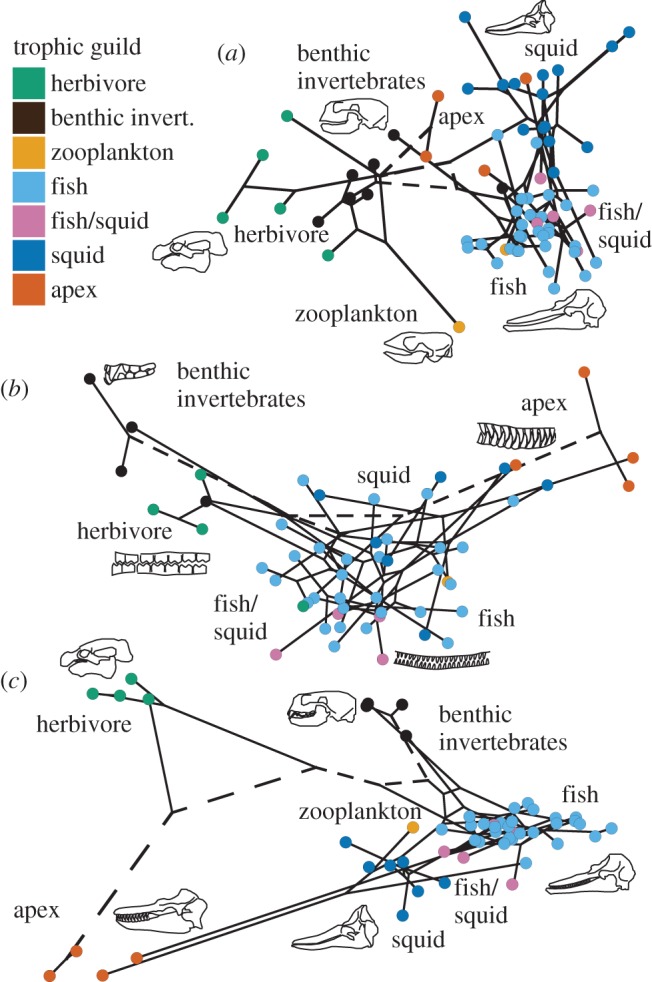
Phylomorphospace using the first two LD axes of three LDAs incorporating (*a*) skull measurements only, (*b*) tooth measurements only, (*c*) both skull and tooth measurements. Colours denote trophic groups (see key); dashed lines indicate divergence prior to marine invasion.

Cranial morphology alone enabled the correct classification of 87% of species across all dietary categories using LDA (figure 2*a*). Discrimination was best among mixed fish and squid feeders (FS) (100% correct classification) and worst among zooplanktivores (Z) (50% correct classification). Classification of all other groups ranged between 75% and 93%. Similarly, tooth measurements alone were able to correctly discriminate dietary groups in 87% of the 54 species included in this analysis with complete dentitions (figure 2*b*). Notably, combined analysis of dentition and cranial measurements achieved the best discrimination, with 100% of all species assigned to the correct dietary group (figure 2*c*).

The first two linear discriminant functions (LD1 and LD2) together explain 68–81% of between-group variance in each LDA (electronic supplementary material, tables S4–S6). In cranial analyses, LD1 was consistently positively influenced by skull length and distance between jaw articulation and posterior tooth (i.e. out-lever for jaw closure at the back of the tooth row), and negatively influenced by jaw depth, rostral breadth, the distance between the jaw articulation and coronoid process (i.e. approximate in-lever for jaw closure), and the distance between the jaw articulation and the anterior tooth (i.e. the out-lever for anterior jaw closure). For skull measurements alone, LD2 was strongly influenced by skull length and posterior skull depth versus rostrum breadth. When tooth measurements were included, LD2 was influenced by rostral length and width relative to skull width, and strongly negatively influenced by jaw length. Linear discriminant functions based on only tooth measurements were influenced by size contrasts between anterior versus posterior and upper versus lower dentition.

## 4. Discussion and conclusion

Previous studies of marine tetrapods have frequently suggested that trophic convergence is reflected in the evolution of marine tetrapod cranial and dental morphology [5,6,14]. While many past investigations have focused on fossil taxa, the results herein represent a quantitative validation of this hypothesis using extant marine tetrapod taxa for which dietary patterns can be directly observed. Trophic guilds resolved from dietary data can be morphologically discriminated across phylogenetically disparate species that include multiple independent marine invasions. We interpret these results to reflect the strong influence of mechanical constraints on food capture and processing modes among marine tetrapods.

One primary distinction that emerged in discriminant analyses is between species that engage in extensive intraoral food processing and those that typically seize and ingest food items whole. This dichotomy resembles Olson's [15] ‘static pressure’ (SP) and ‘kinetic inertial’ (KI) jaw closure modes characterizing tetrapod feeding systems. In this study, herbivores and benthic invertebrate specialists emphasize powerful jaw closure to crop and crush food items prior to ingestion, whereas fish and squid eaters emphasize rapid closure to capture elusive prey that are swallowed with minimal intraoral processing. These contrasts resolve as differences in skull profile and proportions revealed in the discriminant analysis of morphology (figure 2). SP feeders typically have expanded area for jaw muscle attachment and proportionally shorter jaws to increase mechanical advantage. KI feeders possess proportionally longer jaws and lower profile skulls associated with ‘snap feeding’ [14] and potentially increased hydrodynamic efficiency. Apex predators—which capture and dismember large mobile tetrapod prey—employ a combination of these two modes and display intermediate values in the skull-only LDA (figure 2*a*). Notably, these apex predators also consume a substantial fraction of lower-trophic-level prey [9,16], consistent with their versatile feeding morphology.

Investigations of marine mammal feeding modes highlight the distinctions between ram-feeding and suction-feeding species [16–20]. Among cetaceans, these feeding modes are often framed as characterizing fish versus squid specialists, respectively. In our analysis, fish and squid specialists are well discriminated from each other on the basis of skull morphology (figure 2*a*), but not by tooth morphology (figure 2*b*). This may reflect relaxed functional constraint on tooth morphology among squid-eating marine tetrapods [17] or loss of discriminatory power with the exclusion of species with highly reduced dentition. However, suction feeding is variably combined with ram-feeding modes in different cetaceans [18] and is employed by pinnipeds feeding on a variety of prey types [16,20] suggesting that these contrasting feeding modes may not map neatly onto differences in trophic guild. Furthermore, this study did not include hyolingual measurements, an important component in tetrapod suction feeding [7].

Marine tetrapods have long served as canonical examples of convergent evolution, largely based on qualitative comparisons. Linking morphological and ecological datasets within a quantitative framework represents a significant step forward, particularly given our limited understanding of the behaviour and biology of many living marine tetrapod species. The approach outlined here also invites future investigation of the evolution of trophic adaptations in marine tetrapods incorporating extinct species for which direct dietary records are scarce.

## Supplementary Material

Detailed methods, supplementary tables, supplementary figures

## Data accessibility

Electronic supplementary material is available on the Dryad repository (doi:10.5061/dryad.tt36g).
