# Supplementary material for: Trophic convergence drives morphological convergence in marine tetrapods
Source: Biol Lett. 2015 Jan;11(1):20140709. doi: 10.1098/rsbl.2014.0709 (PMC4321144; doi:10.1098/rsbl.2014.0709)
Supplement: Detailed methods, supplementary tables, supplementary figures [file rsbl20140709supp1.doc]

Electronic supplementary material

Trophic convergence drives morphological convergence in marine tetrapods

Neil P. Kelley­1,2*, Ryosuke Motani2

1Department of Paleobiology, National Museum of Natural History, Smithsonian Institution, P.O. Box 37012, Washington, District of Columbia 20013, USA

2Department of Geology, University of California, Davis, 1 Shields Avenue, Davis, California 95616, USA

*corresponding author e-mail address: [kelleynp@si.edu](mailto:kelleynp@si.edu)

Contents

Detailed Methods

Table S1: Diet data and sources

Figure S1: Skull measurements

Table S2: Specimens used in this study

Figure S2: Phylogenetic distribution of diets

Table S3-S6: LDA results

Figure S3: LDA score biplots

**Detailed methods**

**(a) *Dietary Analysis***

Diets of 69 marine mammal and reptile species were tabulated using approximate proportion of a species diet (0-1) comprised by each of the eight dietary categories used by Pauly et al. [S1]: “large zooplankton”; “benthic invertebrates”; “small squid”; “large squid”; “schooling pelagic fish”; “mesopelagic fish”; “mixed fish” (i.e. demersal fish); “high vertebrate” (i.e. tetrapod); see Table S1 for dietary proportions used and literature sources. We included an additional dietary category, “plants” for multicellular primary producers (e.g. seagrasses and algae). We used Ward’s minimum variance hierarchical agglomerative clustering of the Euclidean dissimilarity matrix of this data to search for groupings of species with similar diets. The strength of clusters was evaluated with confidence intervals calculated from 10,000 multiscale bootstrap resampling replicates. Cluster analysis was carried out using R 3.0.3 and bootstrapping *p*-values were calculated using the package pvclust.

**(b) *Morphometric Analysis***

Seventeen skull and jaw measurements were taken from 69 marine and marginal marine tetrapod species. Skull measurements were selected to encompass functionally important aspects of the skull: e.g. width between jaw joints as an approximate measure of pharyngeal size; distance from jaw joint to adductor attachment and distances from jaw joint to anterior and posterior teeth to provide indices of mechanical advantage; see Figure S1 for a complete list of measurements. Twelve tooth measurements were collected for all species except those lacking teeth. Maximum crown height, mesio-distal and labio-lingual diameters were measured at or near the anterior and posterior extreme of the upper and lower tooth rows. We selected fully erupted, functional teeth and avoided vestigial teeth such as are often found at the extreme posterior or anterior ends of tooth rows. The canines of carnivorans and the tusks of *Odobenus* and *Dugong* were excluded.

Measurements were taken with digital calipers (accurate to 0.01 mm). Measurements longer than 300 mm were taken with analog calipers or a tape measure. Although multiple specimens were examined for most species, we included only the single best condition adult specimen for each species in the analyses presented below to avoid differential weighting of species in multivariate analysis. Adult female specimens were used for sexually dimorphic taxa (e.g. pinnipeds). See Table S2 for complete list of specimens used in this study.

We used Linear Discriminant Analysis (LDA) to test the ability of these measurements to discriminate between the dietary categories identified in the cluster analysis. LDA searches for linear combinations of continuous variables (in this case skull measurements) that best separate a collection of individuals into discrete groups (in this case dietary categories). LDA then returns a series of linear functions where the first function explains the largest proportion of between class variance, the next orthogonal function explains the largest proportion of the remaining variance and so on. Class membership can then be “predicted” either to classify individuals whose class membership is unknown, or to compare LDA prediction with known class membership in the training dataset to assess the ability to consistently discriminate classes based on the supplied continuous variables. The latter approach is adopted here to test the ability of dietary groups to be separated based on the linear measurements of skull and tooth dimensions. Individual LDA scores can also be used to visualize group differences (e.g. Figure 2, Figure S3)

All measurements were log-transformed prior to LDA. We calculated LDA scores using three combinations of variables: 1) skull and jaw measurements only; 2) tooth measurements only and 3) all cranial and tooth measurements together. The latter two analyses included fewer species (54 of 69) due to the exclusion of species with reduced or absent dentition (primarily turtles and beaked whales). LDA was conducted using the MASS package in R 3.0.3. Group probabilities (priors) were based on training data.

**(c) *Ancestral State Reconstruction***

A time-calibrated phylogeny of the 69 species included in this analysis was assembled from previously published phylogenetic analyses (see Figure S2) and timetree.org in order to trace the history of trophic diversification within marine tetrapod clades. Trophic groups identified by the cluster analysis were mapped onto this tree and parsimony was used to reconstruct hypothetical dietary habit of internal nodes using Mesquite 2.75. Hypotheses of ancestral trophic diversification were limited to divergences that occurred after the initial invasion of marine environments by a given clade or taxon based on molecular and fossil data as many of the trophic guilds considered here are not applicable to terrestrial ancestors. This tree topology was mapped onto individual LDA scores (explained above) to generate the phylomorphospace shown in Figure 2.

| **Species** | **Diet** | **BI** | **LZ** | **SS** | **LS** | **SP** | **MP** | **MF** | **HV** | **PL** | **Ref.** |
| --- | --- | --- | --- | --- | --- | --- | --- | --- | --- | --- | --- |
| *Amblyrhynchus cristatus* | H | 0 | 0 | 0 | 0 | 0 | 0 | 0 | 0 | 1 | S1 |
| *Arctocephalus galapagoensis* | FB | 0 | 0 | 0.4 | 0 | 0.2 | 0.3 | 0.1 | 0 | 0 | S1 |
| *Arctocephalus pusillus* | FB | 0.1 | 0 | 0.3 | 0.15 | 0.2 | 0 | 0.25 | 0 | 0 | S1 |
| *Arctocephalus townsendi* | FB | 0.2 | 0 | 0.3 | 0 | 0.3 | 0 | 0.2 | 0 | 0 | S1 |
| *Bearardius bairdii* | S | 0.1 | 0 | 0.3 | 0.25 | 0.1 | 0.1 | 0.15 | 0 | 0 | S1 |
| *Callorhinus ursinus* | FB | 0 | 0 | 0.15 | 0.15 | 0.25 | 0.15 | 0.3 | 0 | 0 | S1 |
| *Caretta caretta* | BI | 0.6 | 0 | 0.05 | 0 | 0 | 0 | 0.2 | 0.05 | 0 | S2,S3 |
| *Cephalorhynchus eutropia* | FB | 0.2 | 0 | 0.3 | 0 | 0.3 | 0 | 0.2 | 0 | 0 | S4 |
| *Chelonia mydas* | H | 0.1 | 0.05 | 0 | 0 | 0 | 0 | 0.05 | 0 | 0.8 | S2,S3 |
| *Crocodylus acutus* | G | 0.4 | 0 | 0 | 0 | 0 | 0 | 0.3 | 0.3 | 0 | S5,S6 |
| *Crocodylus porosus* | G | 0.3 | 0 | 0 | 0 | 0 | 0 | 0.4 | 0.3 | 0 | S7,S8 |
| *Cystophora cristata* | FB | 0 | 0 | 0.2 | 0.2 | 0.2 | 0 | 0.4 | 0 | 0 | S1 |
| *Delphinapterus leucas* | FA | 0.2 | 0 | 0.05 | 0.05 | 0.2 | 0.1 | 0.4 | 0 | 0 | S1 |
| *Delphinus capensis* | FB | 0 | 0 | 0.2 | 0.1 | 0.1 | 0.2 | 0.4 | 0 | 0 | S9 |
| *Delphinus delphis* | FS | 0 | 0 | 0.15 | 0.15 | 0.1 | 0.4 | 0.2 | 0 | 0 | S1 |
| *Dermochelys coriacea* | PI | 0 | 1 | 0 | 0 | 0 | 0 | 0 | 0 | 0 | S2,S3 |
| *Dugong dugong* | H | 0.15 | 0 | 0 | 0 | 0 | 0 | 0 | 0 | 0.85 | S10 |
| *Enhydra lutris* | BI | 0.8 | 0 | 0.05 | 0 | 0.05 | 0 | 0.1 | 0 | 0 | S1 |
| *Eretmochelys imbricata* | BI | 0.9 | 0 | 0 | 0 | 0 | 0 | 0 | 0 | 0.1 | S2,S3 |
| *Erignathus barbatus* | BI | 0.65 | 0.15 | 0 | 0 | 0.05 | 0 | 0.15 | 0 | 0 | S1 |
| *Eumetopias jubatus* | FB | 0.15 | 0 | 0.2 | 0.15 | 0.005 | 0 | 0.4 | 0.05 | 0 | S1 |
| *Feresa attenuata* | S | 0 | 0 | 0.3 | 0.2 | 0.1 | 0 | 0.2 | 0.2 | 0 | S1 |
| *Globicephala macrorhynchus* | S | 0 | 0 | 0.3 | 0.3 | 0.1 | 0.1 | 0.2 | 0 | 0 | S1 |
| *Grampus griseus* | S | 0.05 | 0 | 0.5 | 0.35 | 0.05 | 0 | 0.05 | 0 | 0 | S1 |
| *Halichoreus grypus* | FA | 0.15 | 0 | 0.05 | 0 | 0.3 | 0 | 0.45 | 0.05 | 0 | S1 |
| *Histriophoca fasciata* | FA | 0.35 | 0 | 0.1 | 0 | 0.25 | 0 | 0.3 | 0 | 0 | S1 |
| *Hydrurga leptonyx* | G | 0 | 0.35 | 0.1 | 0 | 0.1 | 0 | 0.05 | 0.4 | 0 | S1 |
| *Kogia breviceps* | S | 0.05 | 0 | 0.35 | 0.4 | 0 | 0.1 | 0.1 | 0 | 0 | S1 |
| *Kogia sima* | S | 0.1 | 0 | 0.4 | 0.4 | 0 | 0.05 | 0.05 | 0 | 0 | S1 |
| *Lagenorhynchus obliquidens* | FB | 0 | 0 | 0.3 | 0.05 | 0.3 | 0.2 | 0.15 | 0 | 0 | S1 |
| *Lepidochelys olivacea* | BI | 0.6 | 0.1 | 0 | 0 | 0 | 0 | 0.2 | 0 | 0.1 | S2,S3 |
| *Leptonychotes weddelii* | FA | 0.2 | 0 | 0.15 | 0 | 0.15 | 0 | 0.5 | 0 | 0 | S1 |
| *Lissodelphis borealis* | FS | 0 | 0 | 0.3 | 0.2 | 0 | 0.4 | 0.1 | 0 | 0 | S1 |
| *Lobodon carcinophaga* | PI | 0 | 0.9 | 0 | 0 | 0.1 | 0 | 0 | 0 | 0 | S1 |
| *Lontra felina* | BI | 0.65 | 0 | 0 | 0 | 0.1 | 0 | 0.25 | 0 | 0 | S1 |
| *Mesoplodon carlhubbsi* | S | 0 | 0 | 0.4 | 0.4 | 0 | 0.2 | 0 | 0 | 0 | S1 |
| *Mesoplodon densirostris* | FS | 0 | 0 | 0.2 | 0.3 | 0 | 0.3 | 0.2 | 0 | 0 | S1 |
| *Mesoplodon stejnegeri* | S | 0 | 0 | 0.5 | 0.45 | 0 | 0 | 0.05 | 0 | 0 | S1 |
| *Mirounga angustirostris* | S | 0.05 | 0 | 0.4 | 0.2 | 0 | 0.2 | 0.15 | 0 | 0 | S1 |
| *Mirounga leonina* | S | 0.05 | 0 | 0.4 | 0.35 | 0.05 | 0 | 0.15 | 0 | 0 | S1 |
| *Monachus monachus* | FA | 0.2 | 0 | 0 | 0.2 | 0 | 0.5 | 0.1 | 0 | 0 | S1 |
| *Monachus schauinslandi* | FA | 0.2 | 0 | 0.1 | 0 | 0 | 0 | 0.7 | 0 | 0 | S1 |
| *Monachus tropicalis* | FA | 0.475 | 0 | 0.2 | 0 | 0.1 | 0.05 | 0.125 | 0.05 | 0 | S11 |
| *Neophocoena phoceonoides* | FB | 0.1 | 0 | 0.4 | 0 | 0.2 | 0.1 | 0.2 | 0 | 0 | S1 |
| *Odobenus rosmarus* | BI | 0.85 | 0 | 0 | 0 | 0 | 0 | 0.05 | 0.1 | 0 | S1 |
| *Ommatophoca rossi* | S | 0.05 | 0.15 | 0.5 | 0.15 | 0 | 0 | 0.15 | 0 | 0 | S1 |
| *Orcinus orca* | G | 0 | 0 | 0.05 | 0.05 | 0.1 | 0 | 0.4 | 0.4 | 0 | S1 |
| *Otaria flavescens* | FB | 0.1 | 0.15 | 0.15 | 0.1 | 0.1 | 0 | 0.35 | 0.05 | 0 | S1 |
| *Pagophilus groenlandicus* | FA | 0.1 | 0 | 0.1 | 0.05 | 0.3 | 0 | 0.45 | 0 | 0 | S1 |
| *Peponocephala electra* | S | 0 | 0 | 0.35 | 0.35 | 0.1 | 0.1 | 0.1 | 0 | 0 | S1 |
| *Phoca vitulina* | FA | 0.1 | 0 | 0.1 | 0.05 | 0.3 | 0 | 0.45 | 0 | 0 | S1 |
| *Phocoena phocoena* | FA | 0.05 | 0 | 0.1 | 0.1 | 0.3 | 0 | 0.45 | 0 | 0 | S1 |
| *Phocoena spinipinnis* | FB | 0 | 0.1 | 0.2 | 0.1 | 0.35 | 0 | 0.25 | 0 | 0 | S1 |
| *Phocoenoides dalli* | FB | 0.05 | 0 | 0.3 | 0.1 | 0.2 | 0.2 | 0.15 | 0 | 0 | S1 |
| *Physeter macrocephalus* | S | 0.05 | 0 | 0.1 | 0.6 | 0.05 | 0.05 | 0.15 | 0 | 0 | S1 |
| *Pontoporia blainvillei* | FB | 0.1 | 0 | 0.2 | 0.2 | 0.2 | 0 | 0.3 | 0 | 0 | S1 |
| *Pseudorca crassidens* | S | 0 | 0 | 0.3 | 0.2 | 0.1 | 0 | 0.3 | 0.1 | 0 | S12,S13 |
| *Pusa hipsida* | FA | 0.2 | 0.2 | 0 | 0 | 0.15 | 0.05 | 0.4 | 0 | 0 | S1 |
| *Sotalia fluviatilis* | FA | 0.2 | 0 | 0.1 | 0 | 0.2 | 0 | 0.5 | 0 | 0 | S1 |
| *Stenella attenuata* | FB | 0 | 0 | 0.3 | 0.2 | 0.1 | 0 | 0.4 | 0 | 0 | S1 |
| **Species** | **Diet** | **BI** | **LZ** | **SS** | **LS** | **SP** | **MP** | **MF** | **HV** | **PL** | **Ref.** |
| *Stenella coeruleoalba* | FS | 0.05 | 0 | 0.2 | 0.15 | 0.05 | 0.3 | 0.25 | 0 | 0 | S1 |
| *Stenella longirostris* | FS | 0 | 0 | 0.2 | 0.2 | 0 | 0.4 | 0.2 | 0 | 0 | S1 |
| *Steno brenadensis* | FB | 0.1 | 0 | 0.2 | 0.1 | 0.2 | 0 | 0.4 | 0 | 0 | S1 |
| *Trichechus manatus* | H | 0.1 | 0 | 0 | 0 | 0 | 0 | 0 | 0 | 0.9 | S14,S15 |
| *Trichechus senegalensis* | H | 0 | 0 | 0 | 0 | 0 | 0 | 0 | 0 | 1 | S15 |
| *Tursiops truncatus* | FA | 0 | 0 | 0.2 | 0.05 | 0.15 | 0 | 0.6 | 0 | 0 | S1 |
| *Zalophus californianus* | FB | 0 | 0 | 0 | 0 | 0 | 0 | 0 | 1 | 0 | S1 |
| *Zalophus wollebacki* | FB | 0.1 | 0 | 0.2 | 0.15 | 0.25 | 0 | 0.3 | 0 | 0 | S1 |
| *Ziphius cavirostris* | S | 0.1 | 0 | 0.3 | 0.3 | 0 | 0.15 | 0.15 | 0 | 0 | S1 |

Table S1. Estimated dietary proportions (out of 1) of nine food types for the species included in this investigation. BI - benthic invertebrates, LZ - large zooplankton, SS - small squid, LS - large squid, SP - schooling pelagic, MP - mesopelagic, MF - mixed fish, HV - higher vertebrates (i.e. tetrapod), PL - plants. Categories adapted from Pauly et al. 1998 (see below for complete list of references).

S1. Pauly, D., Trites, A. W., Capuli, E., & Christensen, V. 1998 Diet composition and trophic levels of marine mammals. *ICES J Mar Sci*. **55**, 467-481. (DOI 10.1006/jmsc.1997.0280)

S2. Márquez M. R. 1990. *FAO species catalogue. Vol 11: Sea turtles of the world*. FAO Fisheries

Synopsis No. 125 Vol 11 Rome: FAO

S3. Bjorndal, K. A. 1997. Foraging ecology and nutrition of sea turtles. *The biology of sea turtles*.

CRC Press **1**, 199-231.

S4. Dawson, S. M. 2007. *Cephalorhynchus* Dolphins In: *The Encylcopedia of Marine Mammals*

2nd Edition (eds. Perrin, Würsig and Thewissen), pp. 191-195. Academic Publishing.

S5. Thorbjarnarson, J. 1989. Ecology of the American crocodile, *Crocodylus acutus*. In:

*Crocodiles. Their Ecology, Management and Conservation*. A Special Publication of the

Crocodile Specialist Group*,* pp. 228–258IUCN, Gland, Switzerland.

S6. Villegas, A., & Soto, J. J. S. (2008). Feeding habits of the american crocodile, *Crocodylus*

*acutus* (Cuvier, 1807) (Reptilia: Crocodylidae) in the southern coast of Quintana Roo,

Mexico. *Acta Zoologica Mexicana*, **24**, 117-124.

S7. Allen, G. R. 1974. The marine crocodile, Crocodylus porosus, from Ponape, eastern Caroline

Islands, with notes on food habits of crocodiles from the Palau Archipelago. *Copeia*, **2**, 553-553.

S8. Messel, H., & Vorlicek, G. C. 1989. Ecology of Crocodylus porosus in northern Australia.

*Crocodiles. Their Ecology, Management and Conservation*. A Special Publication of the

Crocodile Specialist Group*,* pp. 164-183.IUCN, Gland, Switzerland

S9. Osnes-Erie, L. D. 1999. Food habits of common dolphin (*Delphinus delphis* and *D. capensis*)

off California. Master’s Thesis. San Jose University.

S10. Preen, A. 1995 Diet of dugongs: are they omnivores? *J. Mammal*. **76**, 163-171. (DOI

10.2307/1382325).

S11. Adam, P. J., & Garcia, G. G. 2003. New information on the natural history, distribution, and

skull size of the extinct (?) West Indian monk seal, *Monachus tropicalis*. Mar. Mammal science, **19**, 297-317.

S12. Baird, R. W. 2007. False Killer Whale In: *The Encylcopedia of Marine Mammals* 2nd Edition

(eds. Perrin, Würsig and Thewissen), pp. 405-406. Academic Publishing.

S3. Alonso, M. K., Pedraza, S. N., Schiavini, A., Goodall, R. N. P., and Crespo, E. A. 1999.

Stomach contents of false killer whales (*Pseudorca crassidens*) stranded on the coasts of the Strait of Magellan, Tierra del Fuego. *Mar. Mamm. Sci.* **15**, 712-724.

S14. Courbis, S. S., and Worthy, G. A. (2003). Opportunistic carnivory by Florida manatees

(*Trichechus manatus latirostris*). *Aquatic Mammals*. **29**, 104-107.

S15. Reynolds, J. E., Powell, J. A. and Taylor C. R. 2007. Manatees. In: *The Encylcopedia of Marine*

*Mammals* 2nd Edition (eds. Perrin, Würsig and Thewissen), pp 682-692 Academic

Publishing.

**
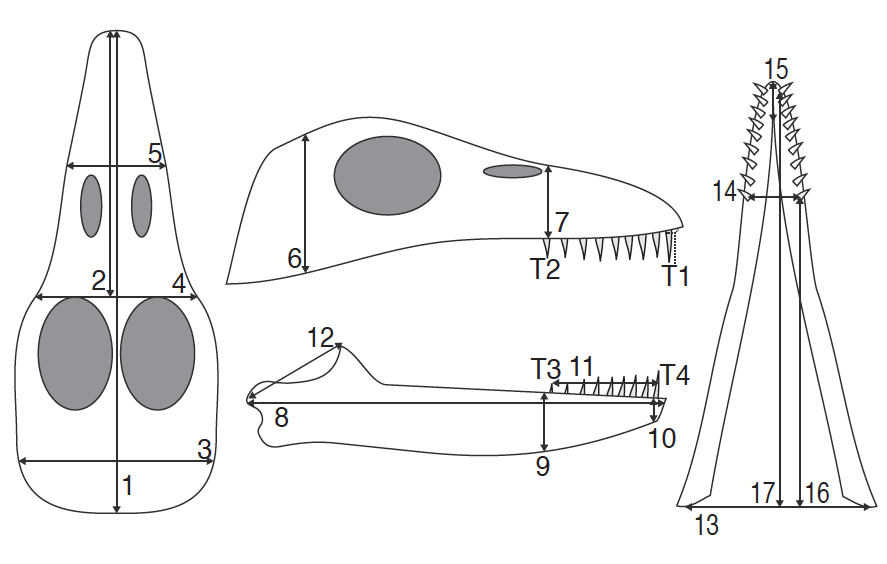
**

Figure S1. Schematic skull diagram of a hypothetical generalized aquatic tetrapod showing 17 skull measurements and 12 tooth measurements used in the morphometric analyses:

1) Skull length: distance between the inion and prosthion.

2) Rostrum length: distance between anterior margin of orbit and prosthion.

3) Maximum postorbital skull width.

4) Antorbital skull width at the anterior margin of the orbit.

5) Mid-rostral width at ½ distance from anterior plane of orbit to the prosthion (2).

6) Maximum postorbital skull height.

7) Mid-rostral skull height: measured perpendicular to 5.

8) Jaw length: from the articular condyle to the gonion.

9) Jaw depth at posterior margin of tooth row*.

10) Jaw depth at mandibular symphysis.

11) Lower tooth row length*.

12) Distance from articular condyle to tip of coronoid process.

13) Width at midpoint of articular condyles.

14) Width of lower jaw at posterior toothrow*.

15) Length of mandibular symphysis.

16) Distance from plane of jaw articulation to posteriormost tooth*.

17) Distance from plane of jaw articulation to anteriormost tooth*

T1-T4 approximate location of teeth measured on each skull. For each tooth, maximum crown height, labio-lingual width, and mesio-distal length were recorded. For multicusped teeth, crown height was measured as base of tooth crown to tallest cusp.

* - *For turtles measured from analogous position on triturating surface.*

| **Taxon** | **Specimen** |  | **Taxon** | **Specimen** |
| --- | --- | --- | --- | --- |
| *Amblyrhynchus cristatus* | MVZ67708 |  | *Mesoplodon carlhubbsi** | CAS24811 |
| *Arctocephalus galapagoensis* | CAS20833 |  | *Mesoplodon densirostris** | CAS22924 |
| *Arctocephalus pusillus* | CAS6040 |  | *Mesoplodon stejnegeri** | CAS16596 |
| *Arctocephalus townsendi* | CAS23838 |  | *Mirounga angustirostris* | CAS15925 |
| *Bearardius bairdii** | CAS27551 |  | *Mirounga leonina* | CAS6251 |
| *Callorhinus ursinus* | CAS23825 |  | *Monachus monachus* | NMNH219059 |
| *Caretta caretta** | MVZ228794 |  | *Monachus schauinslandi* | WFB7323 |
| *Cephalorhynchus eutropia* | MVZ18854 |  | *Monachus tropicalis* | CAS4978 |
| *Chelonia mydas** | MVZ222408 |  | *Neophocoena phoceonoides* | NMNH241503 |
| *Crocodylus acutus* | MVZ222426 |  | *Odobenus rosmarus* | CAS261 |
| *Crocodylus porosus* | MVZ81487 |  | *Ommatophca rossi* | NMNH270321 |
| *Cystophora cristata* | ROM1666 |  | *Orcinus orca* | CAS16464 |
| *Delphinapterus leucas* | MVZ123976 |  | *Otaria flavescens* | CAS16229 |
| *Delphinus capensis* | CAS25533 |  | *Pagophilus groenlandicus* | ROM1665 |
| *Delphinus delphis* | CAS16336 |  | *Peponocephala electra* | NMNH504250 |
| *Dermochelys coriacea** | MVZ226847 |  | *Phoca vitulina* | CAS5 |
| *Dugong dugong* | NMNH22481 |  | *Phocoena phocoena* | CAS27231 |
| *Enhydra lutris* | WFB2115 |  | *Phocoena spinipinnis* | CAS16179 |
| *Eretmochelys imbricata** | MVZ208244 |  | *Phocoenoides dalli* | CAS24491 |
| *Erignathus barbatus* | NMNH396801 |  | *Physeter catadon** | MVZ140475 |
| *Eumetopias jubatus* | CAS13818 |  | *Pontoporia blainvillei* | CAS15256 |
| *Feresa attenuata* | NMNH504916 |  | *Pseudorca crassidens* | NMNH484982 |
| *Globicephala macrohynchus* | MVZ97812 |  | *Pusa hipsida** | CAS10166 |
| *Grampus griseus** | CAS24336 |  | *Sotalia fluviatilis* | CAS16658 |
| *Halichoreus grypus* | CAS22571 |  | *Stenella attenuata* | CAS23247 |
| *Histriophoca fasciata* | CA16614 |  | *Stenella coeruleoalba* | CAS16720 |
| *Hydruga leptonyx* | MVZ127753 |  | *Stenella longirostris* | CAS15668 |
| *Kogia breviceps** | CAS28125 |  | *Steno brenadensis* | MVZ175111 |
| *Kogia sima** | CAS16635 |  | *Trichechus manatus* | CAS24787 |
| *Lagenorhynchus obliquidens* | MVZ191014 |  | *Trichechus senegalensis* | MVZ4822 |
| *Lepidochelys olivacea** | MVZ200233 |  | *Tursiops truncatus* | WFB2828 |
| *Leptonychotes weddeli* | MVZ127756 |  | *Zalophus californianus* | CAS433 |
| *Lissodelphis borealis* | CAS25549 |  | *Zalophus wollebacki* | CAS1190 |
| *Lobodon carcinophaga* | MVZ127751 |  | *Ziphius cavirostris** | CAS13482 |
| *Lontra felina* | NMNH25168 |  |  |  |

Table S2: Specimens included in this study. Birds, baleen whales (Mysticeti) and sea snakes (Hydrophiinae, Laticaudinae) were not considered in the present analysis due to concerns that morphological and functional novelties of these groups could obscure ecologically important morphological differences among the studied species.

* - Taxa excluded from analyses including tooth measurements due to absence of compete dentition in species or specimen.

Museum abbreviations:

**CAS** -California Academy of Sciences, San Francisco, CA, USA

**MVZ** - Museum of Vertebrate Zoology, University of California (UC) Berkeley, CA, USA

**NMNH** - National Museum of Natural History, Washington DC, USA

**ROM** - Royal Ontario Museum, Toronto, ON, Canada

**WFB** - Wildlife and Fisheries Museum, UC Davis, CA, USA

Figure S2: Time-calibrated phylogeny of species included in this analysis. Divergence ages are calculated from Timetree.org[1]. Arrows mark approximate entrance timing of major clades and stars mark approximate entrance timing of minor lineages based on fossil and molecular clock data. Branch color indicates diet (see key) with hypothetical ancestral reconstruction made using parsimony with Mesquite 2.75. Grey nodes and branches are equivocal. Nodes predating marine entrance were precluded from dietary reconstruction, as many marine dietary categories are inapplicable for terrestrial ancestors.

1. Hedges S. B., Dudley J., & Kumar S. (2006). TimeTree: A public knowledge-base of

divergence times among organisms. *Bioinformatics* **22**: 2971-2972.

| **Measurement** | **LD1** | **LD2** | **LD3** | **LD4** | **LD5** | **LD6** |
| --- | --- | --- | --- | --- | --- | --- |
| **1** | 10.08 | -13.04 | -3.84 | -0.44 | 7.49 | 7.24 |
| **2** | 3.19 | -4.03 | -6.21 | 1.21 | 4.84 | 4.08 |
| **3** | -1.63 | 7.75 | -2.28 | 5.39 | -1.29 | -5.65 |
| **4** | -10.04 | 3.76 | -6.36 | 0.44 | -7.2 | 4.24 |
| **5** | 1.89 | 1.02 | 4.83 | -4.41 | 3.27 | -1.39 |
| **6** | -1.03 | -6.37 | -10.31 | -7.83 | -1.42 | -3.49 |
| **7** | 2.91 | -1.92 | 1.59 | 2 | 2.76 | -1.51 |
| **8** | -7.12 | -0.14 | 31.51 | -11.69 | -14.5 | -4.3 |
| **9** | -6.95 | 3.67 | -2.04 | -4.55 | -6.08 | -5.72 |
| **10** | 1.2 | -2.36 | -0.37 | 3.26 | 0.54 | 4.25 |
| **11** | 4.36 | -2.11 | 1.18 | 1.76 | 2.16 | -0.22 |
| **12** | -6.46 | 1.26 | 0.95 | 0.28 | 4.16 | 3.32 |
| **13** | 7.23 | 3.98 | 0.12 | 1.73 | 2.46 | 1.22 |
| **14** | 3.54 | -4.61 | 3.99 | 6.71 | 2.19 | 1.23 |
| **15** | -2.48 | 1.55 | -2.63 | 0.24 | 0.53 | -2.03 |
| **16** | 11.64 | 3.27 | -1.98 | 2.36 | 5.71 | -1.05 |
| **17** | -7.39 | 9.64 | -5.36 | 6.67 | -5.81 | -0.66 |
|  |  |  |  |  |  |  |
| ***Proportion of group variance*** | *0.44* | *0.24* | *0.24* | *0.09* | *0.07* | *0.03* |

Table S3. Linear discriminant function scaling and proportion of group variance explained by each function for skull measurements LDA. Numbered measurements correspond to skull measurements illustrated in Figure S1.

| **Measurement** | **LD1** | **LD2** | **LD3** | **LD4** | **LD5** | **LD6** |
| --- | --- | --- | --- | --- | --- | --- |
| **LPCH** | -0.79 | 4.69 | -2.94 | -2.73 | -1.9 | 2.58 |
| **LPCW** | -5.22 | -11.04 | -1.86 | -2.16 | -9.79 | 1.01 |
| **LPCL** | 5.11 | -4.21 | 7.16 | 0.71 | 10.46 | -1.61 |
| **LACH** | 4.36 | 0.98 | 1.22 | -2.41 | -1.96 | -4.96 |
| **LACW** | 3.32 | -1.82 | -0.18 | -2.5 | 0.4 | 1.42 |
| **LACL** | -6.37 | -0.39 | 6 | 3.25 | 0.76 | -1.89 |
| **UPCH** | 3.87 | -4.84 | -4.79 | 7.06 | 5.32 | -1.07 |
| **UPCW** | -8.9 | 7.16 | 3.2 | 1.28 | 1.85 | -2.69 |
| **UPCL** | 4.04 | 6.46 | -9.63 | -6.47 | -3.17 | 0.16 |
| **UACH** | 2.07 | 1.22 | 2.65 | -1.23 | -1.45 | 3.29 |
| **UACW** | 2.49 | 1.7 | 2.84 | 1.65 | -0.83 | 5.87 |
| **UACL** | -1.03 | 2.48 | -5.41 | 3.99 | 1.87 | -2.57 |
|  |  |  |  |  |  |  |
| ***Proportion of group variance*** | ***0.47*** | ***0.22*** | ***0.13*** | ***0.11*** | ***0.05*** | ***0.02*** |

Table S4. Linear discriminant function scaling and proportion of group variance explained by each function for analysis of tooth measurements only. Tooth measurements are as follows: LPCH - lower posterior crown height, LPCW - lower posterior crown width (labio-lingual), LPCL - lower posterior crown length (mesio-distal), LACH - lower anterior crown height, LACW - lower anterior crown width, LACL - lower anterior crown length, UPCH - upper posterior crown height, UPCW – upper posterior crown width, UPCL - upper posteior crown length, UACH - upper anterior crown height, UACW - upper anterior crown width, UACL - upper anterior crown length.

| **Measurement** | **LD1** | **LD2** | **LD3** | **LD4** | **LD5** | **LD6** |
| --- | --- | --- | --- | --- | --- | --- |
| **1** | -23.8 | -8.21 | 2.2 | -7.05 | 12.04 | -7.51 |
| **2** | -6.41 | 22.24 | 9.91 | 6.1 | 0.35 | 11.08 |
| **3** | 19.32 | -3.7 | 1.76 | 0.27 | 7.27 | 9.42 |
| **4** | 22.88 | 0.48 | -13.65 | -0.09 | 1.74 | 1.26 |
| **5** | -13.2 | 4.39 | 18.11 | -1.3 | 6.21 | -8.19 |
| **6** | -17.66 | 15.64 | -7.73 | 8.15 | -1.5 | 4.08 |
| **7** | -8.06 | 1.62 | 3.91 | 0.43 | -3.86 | 0.2 |
| **8** | 15.36 | -28.6 | -23.98 | 10.05 | -18.99 | -17.88 |
| **9** | 27.74 | -3.69 | -4.52 | 0.01 | 5.23 | -2.95 |
| **10** | -6.4 | 0.67 | -1.87 | 4.46 | -3.25 | 5.7 |
| **11** | -11.84 | -3.46 | 10.93 | 2.83 | 1.15 | -3.26 |
| **12** | 8.36 | 12.34 | 8.33 | 9.27 | -0.87 | -0.54 |
| **13** | -5.12 | -2.14 | 6.11 | -16.39 | -9.91 | -5.94 |
| **14** | -10.58 | -12.52 | -7.92 | 1.81 | -8.66 | 10.12 |
| **15** | -0.17 | 2.11 | 4.19 | 0.08 | -1.77 | 2.53 |
| **16** | -17.11 | -11.22 | 3.81 | -9.96 | 1.78 | -0.68 |
| **17** | 25.86 | 1.13 | -3.43 | -2.32 | 10.34 | 2.4 |
| **LPCH** | 4.11 | 4.93 | 8.79 | -5.04 | -1.83 | 2.58 |
| **LPCW** | -1.38 | 10.35 | -1.52 | 9.25 | 5.64 | -5.43 |
| **LPCL** | -9.35 | -16.61 | -9.31 | 0.27 | -6.66 | 0.33 |
| **LACH** | 3.56 | -10.47 | -4.42 | -0.31 | -2.97 | -1.3 |
| **LACW** | 2.75 | 1.4 | 2.09 | 2.47 | 0.44 | -2.41 |
| **LACL** | -11.51 | 3.37 | -5.73 | -8.69 | -3.85 | -3.57 |
| **UPCH** | -2.05 | -6.84 | -10.59 | 3.62 | 7.04 | 7.04 |
| **UPCW** | -11.55 | 8.64 | 2.35 | -13.28 | -4.76 | -2.16 |
| **UPCL** | 22.17 | 2.77 | 16.85 | 12.55 | 5.86 | 0.6 |
| **UACH** | 4.29 | -2.43 | 0.81 | -1.13 | -1.8 | -2.24 |
| **UACW** | 1.66 | -2.99 | -3.5 | -3.12 | -3.28 | 6.14 |
| **UACL** | 2.3 | 5.38 | 0.28 | -0.76 | 8.59 | 0.61 |
|  |  |  |  |  |  |  |
| ***Proportion of group variance*** | ***0.42*** | ***0.39*** | ***0.07*** | ***0.06*** | ***0.04*** | ***0.02*** |

Table S5. Linear discriminant function scaling and proportion of group variance explained by each function for analysis of combined skull and tooth measurements. Numbered measurements correspond to skull measurements illustrated in Figure S1. Acronyms correspond to tooth measurements described on Table 2.4

|  | **Percent correct classification (ncorrect/ntotal)** | | |
| --- | --- | --- | --- |
| **Diet Category** | **Skull only** | **Tooth only** | **Skull + tooth** |
| Herbivore (H) | 80% (4/5) | 75% (3/4) | 100% (4/4) |
| Benthic invertebrates (BI) | 86% (6/7) | 100% (4/4) | 100% (4/4) |
| Planktonic invertebrates (PI) | 50% (1/2) | 100% (1/1) | 100% (1/1) |
| Fish (F) | 87% (27/31) | 97% (29/30) | 100% (30/30) |
| Fish/Squid (FS) | 100% (5/5) | 75% (3/4) | 100% (4/4) |
| Squid (S) | 93% (14/15) | 42.9% (3/7) | 100% (15/15) |
| Apex/General (G) | 75% (3/4) | 100% (4/4) | 100% (4/4) |
| *Total* | *87% (60/69)* | *87% (47/54)* | *100%*  (54/54) |

Table S6. Post-hoc classification accuracy for linear discriminant analyses.


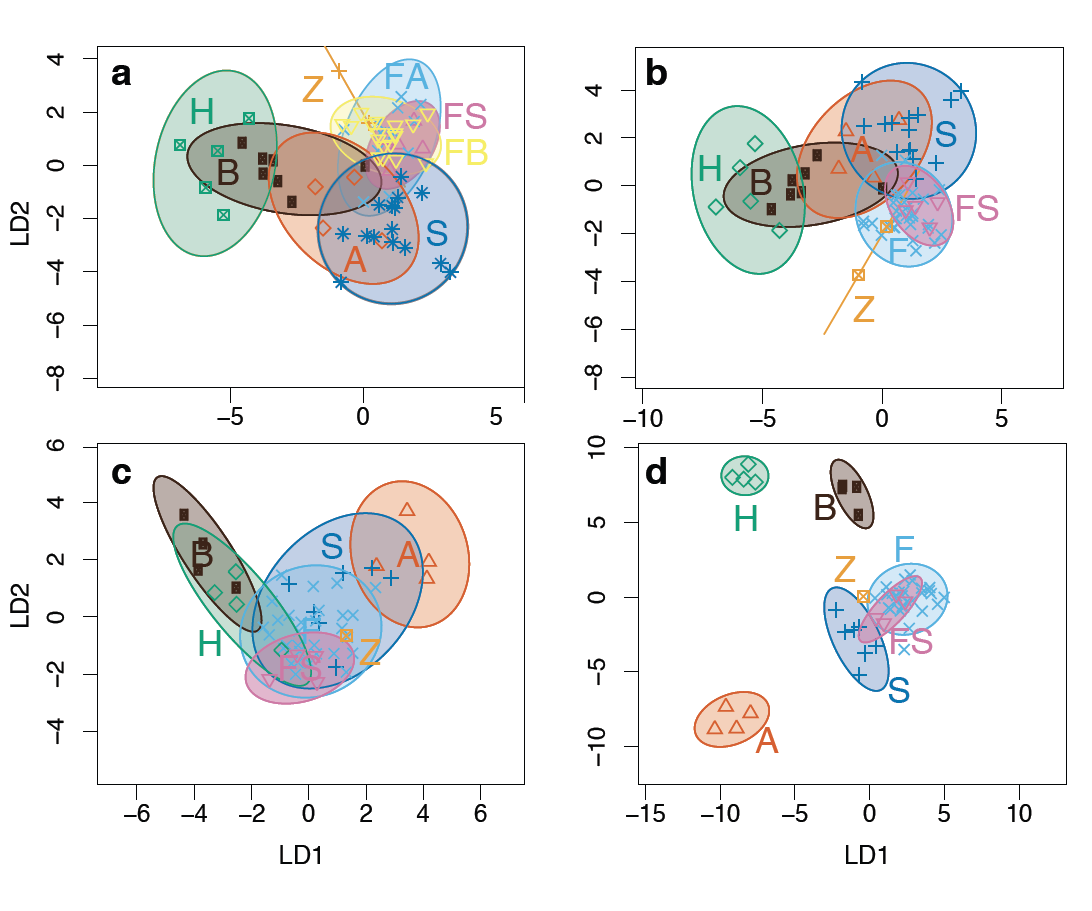


Figure S3. Linear discriminant analysis of skull and tooth measurements a) First two LD axes of LDA incorporating skull measurements 69 species of marine tetrapod in eight dietary categories. b) Same as 3a but with two fish-dominated diet categories (FA and FB) combined. c) First two LD axes of LDA based on tooth measurements alone, same categories as in 3b. d) First two LD axes of LDA based on skull and tooth measurements combined, same categories as 3b and 3c. 3c and 3d include a reduced subset of 54 species with complete upper and lower dentition. Dietary category abbreviations are explained in text.
